# HOME MAINTENANCE STRESS AND BURDEN IN A SAMPLE OF INFORMAL CAREGIVERS OF PERSONS WITH DEMENTIA

**DOI:** 10.1093/geroni/igae098.4207

**Published:** 2024-12-31

**Authors:** Patricia Griffiths, Louis Tennenbaum, Seymour Turner, Susan Kimmel, Jon Sanford

**Affiliations:** PDGRIFF, LLC, Blairsville, Georgia, United States; Homes Renewed Ventures, Washington, DC, District of Columbia, United States; HomesRenewed Ventures, Washington, District of Columbia, United States; Homes Renewed Ventures, Washington, DC, District of Columbia, United States; Georgia State University, atlanta, Georgia, United States

## Abstract

Home maintenance (HM) as it relates to stress, burden and aging in place (AiP) of informal caregivers (ICGs) of persons with dementia (PwD) is undervalued and understudied, yet a crucial component of a safe and reliable home environment. Homes Renewed Ventures surveyed a convenience sample of 14 ICGs caring for PwDs to assess stress and burden associated to HM and the relationship between HM, disruptions to, and AIP. HM Stress and burden were rated on a 5-point scale (none to a great deal) and were highly correlated (r=.92). ICGs reported high stress (mean = 3.9 + 1.4) and burden (3.6 + 1.5) in relation to HM (median and mode stress = 5, burden 4 and 5). Almost three-quarters (73%) had HM problems that disrupted daily routines with five instances resulting in relocations. ICGs rated the likelihood of doing more HM if provided with information (a) customized to their home or (b) on who to call and financing. The means, modes and medians were four or more indicating a high likelihood to do more HM if provided with helpful, personalized information. A Fisher’s exact test indicated a significant association (p=.027) between HM Stress and the likelihood of doing more if provided with option a) customized information about what needs to be done. Although a small convenience sample, these preliminary findings provide strong evidence that HMI are disruptive and stressful, suggesting that enhancing caregiver mastery of these issues is a potential mechanism for reducing ICG stress and burden.

